# Levels of Rare Earth Elements in Food and Human Dietary Exposure: A Review

**DOI:** 10.1007/s12011-024-04297-z

**Published:** 2024-07-06

**Authors:** Neus González, Jose L. Domingo

**Affiliations:** School of Medicine, Laboratory of Toxicology and Environmental Health, Universitat Rovira i Virgili, 43201 Reus, Catalonia Spain

**Keywords:** Rare earth elements (REEs), Human exposure, Foodstuffs, Dietary intake, Health risk

## Abstract

Rare earth elements (REEs) are a group consisting of the following 17 metals: Ce, Dy, Er, Eu, Gd, Ho, La, Lu, Nd, Pr, Pm, Sc, Sm, Tb, Tm, Y and Yb. In the current century, the number of applications of REEs has significantly increased. They are being used as components in high technology devices of great importance industrial/economic. However, information on the risk of human exposure to REEs, as well as the potential toxic effects of these elements is still limited. In general terms, dietary intake is the main route of exposure to metals for non-occupationally exposed individuals, which should be also expected for REEs. The current paper aimed at reviewing the studies -conducted over the world- that focused on determining the levels of REEs in foods, as well as the dietary intake of these elements. Most studies do not suggest potential health risk for consumers of freshwater and marine species of higher consumption, or derived from the intake of a number of vegetables, fruits, mushrooms, as well as other various foodstuffs (honey, tea, rice, etc.). The current estimated daily intake (EDI) of REEs does not seem to be of concern. However, considering the expected wide use of these elements in the next years, it seems to be clearly recommendable to assess periodically the potential health risk of the dietary exposure to REEs. This is already being done with well-known toxic elements such as As, Cd, Pb and Hg, among other potentially toxic metals.

## Introduction

Rare earth elements (REEs) belong to the category of emerging contaminants associated with new technologies. According to the International Union of Pure and Applied Chemistry (IUPAC), REEs consist of lanthanum (La) and the following elements: cerium (Ce), dysprosium (Dy), erbium (Er), europium (Eu), gadolinium (Gd), holmium (Ho), lutetium (Lu), neodymium (Nd), praseodymium (Pr), promethium (Pm), samarium (Sm), terbium (Tb), thulium (Tm) and ytterbium (Yb), as well as scandium (Sc) and yttrium (Y) [66, 67]. REEs are usually divided into heavy rare earth elements (HREEs), which include Gd, Tb, Dy, Ho, Er, Tm, Yb, Lu, Sc and Y, and light rare earth elements (LREEs), which include La, Ce, Pr, Nd, Pm, Sm and Eu [1, 74]. REEs are currently playing an increasing important role in electronics, defense, aerospace, clean energy and automotive, among other relevant industrial sectors. In 2021, manufacturing permanent magnets was the largest and most important end use for REEs. It accounted for 43% of the demand (USGS, 2023 a,b).

In the past century, some REEs were already being used in industrial activities and research. For example, in the 1970s and 1980s, battery research led to the development of the nickel–metal hydride battery, which used La and Nd. Other REEs were also used as catalysts, in magnets, as well as in steel alloys. Since the world society has recognized the critical, specialized properties of REEs, which contribute to the modern technology, in the current century REEs have been increasing their visibilities through many media [48]. It is well-known that REEs have specific physical and chemical properties, such as unique magnetic and optical properties. It means that REEs have a considerable number of applications that involve many aspects of the modern life. Since the late 1990s, and currently, China has globally the dominance in the production and supply of REEs, providing 85 –95% of the world’s REEs [1, 66, 67]. Nowadays, REEs are used as components in a wide number of high technology devices. Some well-known examples are computer monitors and hard disks, smart phones, digital cameras, flat screen televisions, fluorescent and light-emitting-diode lights, and electronic displays, among others. Although the use of REEs in this wide range of electronic devices means only a small part of the final product, these cannot function without them (Versa [68]). Therefore, the use of REEs in electronics is currently essential, not having any substitute. In addition, their use in versatile industries makes them even more crucial.

Despite the great and increasing interest that REEs are having in our modern societies, the global environmental effects, the potential environmental risk associated with the development of REEs production, the REEs toxicities, or the potential adverse human health effects of REEs are issues of considerable importance on which the current information is rather scarce [8, 26, 45–47, 75]. For non-occupationally exposed individuals, it is well established that the diet is -in general- the main source of human exposure to metals [23, 69]. Consequently, to know the dietary intake of REEs should be an issue of notable interest. The objective of the current paper was to review and summarize the results -available in the scientific community- of the studies concerning the concentrations of REEs in foods, as well as their dietary intake, when data were reported. Those studies conducted by regional, national, or international agencies, and/or food safety authorities, are not included in the present review. We have here used the databases PubMed (https://pubmed.ncbi.nlm.nih.gov/) and Scopus (https://www.scopus.com/). The terms used for the search were the following: “rare earth elements”, “REEs”, “food”, “dietary intake” and “human exposure”. Information on the available studies and their results are next summarized.

## Levels of REEs in Freshwater and Marine Organisms

It is well-known that fish, and especially marine fish, constitute an important part of the human diet. This means that its quality and its safety aspects are issues of interest [12, 13, 51]. For their health benefits, people worldwide consume fish and seafood, which provide vitamins and minerals, proteins, low saturated fat content, and omega fatty acids, among other important nutrients [13, 14, 24, 44]. In contrast to these benefits, various investigations have shown that fish and seafood consumption could lead to exposure to a variety of chemical contaminants, which might cause harm to the human body [14, 44]. In recent years, the concentrations of metals in a number of species of fish and seafood have been measured in various areas/zones around the world [23, 25, 33, 50]. However, studies regarding the concentrations of REEs in freshwater and marine organisms are much more limited. These studies are reviewed here.

Table 1 summarizes the studies that focused on determining the levels of REEs in freshwater and marine organisms. Most of these studies were conducted in China. Thus, Zhang et al. [77] measured the concentrations of 14 REEs in sixty samples of marine organisms, which were collected at six sampling sites in the Shenzhen coastal region. The mean concentrations of total REEs were 2.66 ± 2.28, 4.69 ± 1.32, 0.03 ± 0.03 and 0.07 ± 0.02 µg/g, for mollusk, barnacle, shrimp, and fish, respectively. An enrichment of the LREEs with respect to HREEs was observed in all marine organisms. Also in China, Yang et al. [73] carried out a survey that aimed at comparing the concentrations of 14 REEs in freshwater and marine fish of Shandong Province (Eastern of China). The concentrations of 14 REEs were analyzed in muscle samples of four freshwater fish and six marine fish, which were obtained from local markets and supermarkets within 17 cities of the Shandong Province. The selected fish species were the most commonly consumed by the Shandong population. It was found that freshwater fish had a relatively higher ΣREEs than marine fish (34.0–37.9 μg/kg wet weight (ww) vs. 12.7–37.6 μg/kg ww). The assessment of the potential human health risk indicated that fish would mean little risk derived from exposure to REEs through fish consumption. In order to assess the potential impacts of the REEs industry, Ma et al. [38] collected samples of water and suspended particles in the Pearl River Delta (China), as well as oysters of the same place. The levels of 14 REEs were determined in all samples. The sum of REEs concentrations in the Pearl River Delta oysters ranged between 1.8–9.1 and 0.9–2.6 μg/g for the samples collected in December 2016 and June 2017, respectively. It was concluded that in the Pearl River Delta, the dominant REEs uptake pathway in oysters was derived from particles. In turn, in the same Shandong Province, Jiao and co-workers (2021) measured the levels of 14 REEs in 120 commercial mantis shrimp (*O*. *Oratoria)* samples, which were collected from the coast of three sites of Shandong Peninsula. The mean concentration of ΣREEs was 25.1 ng/g (range: 9.04–75.1 ng/g). The levels of REEs reported by Jiao et al. [32] were similar to those previously found for marine fish (12.7 to 37.6 ng/g) by Yang et al. [73], but lower than those found for Pearl River Delta oysters (1.8 to 9.1 μg/g) by Ma et al. [38]. The mean concentrations of the 14 analyzed REEs in *O*. *oratoria* followed the following decreasing order: La > Ce > Nd > Pr > Gd > Sm > Dy > Er > Yb > Eu > Tb > Ho > Tm > Lu. Recently, Wang et al. [72] determined the concentrations of 14 REEs in 14 marine wild fish species from the northern coastal region of the South China Sea. The distribution patterns were characterized and the potential human health risk of these 15 REEs was also assessed. The ∑REEs ranged between 1.02 and 178.55 μg/kg, with a mean concentration of 27.14 μg/kg. The highest average value of ∑REEs was found in *Apogon quadrifasciatus*, while the lowest levels corresponded to *Ariomma indica*. The estimated daily intake (EDI) of REEs through fish consumption was significantly lower than the recommended EDI limit. It clearly indicated that human exposure to REEs through fish consumption of that area would be negligible.

**Table 1:** A summary of REE levels in freshwater and marine species

Region/country	Analyzed samples	Analyzed REEs	Mean values and/or ranges	Remarks	Reference
Shenzen coastal region, China	mollusk, barnacle, shrimp, fish	14	∑REEs: 2.26, 4.69, 0.03 and 0.07 µg/g, in mollusk, barnacle, shrimp and fish, respectively	Higher levels of LREEs than HREEs	Zhang et al. [77]
Shandong Province, China	freshwater (4) and marine (6) species	14	∑REEs: 34.0–37.9 µg/kg (freshwater species); 12.7–37.6 µg/kg (marine species)	No health risk derived from fish consumption	Yang et al. [73]
Pearl River Delta, China	oysters	14	∑REEs: 1.8–9.1 µg/g (2016 sampling); 0.9–2.6 µg/g (2017 sampling)	No specific remarks	Ma et al. [38]
Shandong peninsula, China	shrimps	14	∑REEs. 25.1 (9.04–75.1) µg/kg	La and Ce showed the highest levels	Jiao et al. [32]
Northern region of the South China Sea, China	14 marine wild fish species	15	∑REEs: 27.14 (1.02–178.55) µg/kg	The highest levels of ∑REEs were found in *Apogon quadrifascatius*	Wang et al. [72]
Northwestern Mediterranean Sea, Italy	Fish, mussels, oysters	La and Ce	Fish levels < LOD for La and Ce; mussels, 37 and 65 µg/kg, for La and Ce; oysters, 12 and 20 µg/kg, for La and Ce	No specific remarks	Squadrone et al. [59]
Northwestern Mediterranean Sea, Italy (3 sites)	Brown, green and red seaweeds	16	∑REEs: 7.9, 8.5 and 22, for the different 3 sampling sites	No specific remarks	Squadrone et al. [60]
Ligurian Sea, Italy	fish, bivalves, seaweeds and zooplankton	16	∑REEs: fish (0.21 mg/kg); bivalves (0.16 mg/kg; seaweeds (12 mg/kg) and zooplankton (0.12 mg/kg)	A periodical monitoring of the REEs levels in fish and bivalves was recommended	Squadrone et al. [61]
Italian aquarium	Spotted dogfish (samples of blood, liver, muscle and kidney	16	∑REEs: 4.1, 30, 15 and 13 µg/kg, for blood, liver, muscle and kidney	No specific remarks	Squadrone et al. [64]
Tokyo Bay, Japan	5 species of bivalves (samples of shells and soft tissues)	14	The individual concentrations of the 14 analyzed REEs in shells and soft tissues are all reported in the original paper	REEs levels in soft tissues were higher than those found in shells	Akagi and Edanami [2]
Six sampling areas in the state of Washington, USA	12 freshwater fish species	16	∑REEs: 243 µg/kg (dry weight) (14–3000 µg/kg dw)	The highest ∑REEs levels were found in benthic feeding species of fish	Mayfield and Fairbrother [39]
14 Canadian lakes	abiotic and food web components	15	Results are available in the original paper	The ∑REEs levels in benthic invertebrates was 1000 times higher than those found in fish muscles	Aymot et al. (2017)
31 sampling stations along the French shoreline, France	mussels and oysters	14	∑REEs: 0.18–4.05 µg/g for mussels and 0.21–10.94 µg/g for oysters	The highest concentrations (for oysters) were found in the Gironde estuary	Briant et al. [7]
Gdansk Bay, Poland and North-East Atlantic, Spain	Baltic herring and sardines	17	∑REEs; samples of muscles: 76 µg/kg for herring and 191 µg/kg for sardine	No specific remarks	Reindl and Falkowska, [52]
Six locations of the Portuguese coast, Portugal	mussels	15	∑LREEs: 288–2160 µg/kg ∑HREEs: 54–230 µg/kg	No specific remarks	Figueiredo et al. [22]
Black Sea, Crimea	mussels	16	∑REEs: 710 µg/kg (dw)	In soft tissue, La and Ce, followed by Nd, Sc and Gd were the predominant REEs	Chelyadina et al. [9]
Subaé River, Todos os Santos Bay, Brazil	samples of food web (fish, shellfish, phyto- and zooplankton	15	∑REEs: 6.01, 1.22 and 0.059 mg/kg for bivalves, crustacean and fish	The lowest REEs contents were found in fish muscle	Santos et al. [55]
Southern Caspian Sea, Iran	Golden grey mullet	16	∑REEs: 14.5 µg/g	The relationship between ∑HREEs and ∑LREEs was 1.53	Bakhshalizadeh et al. [4]

Italy is another country in which a number of studies on this same topic have been also performed. The presence of REEs in marine species has been extensively investigated in the Istituto Zooprofilattico Sperimentale del Piemonte, Liguria e Valle d'Aosta, Turin. Squadrone et al. [59] determined the concentrations of 21 trace elements in fish (*Dicentrarchus labrax*), mussels and oysters, which were collected from aquaculture marine ecosystems of the NW Mediterranean Sea. Among these 21 elements, two REEs, Ce and La, were included. These REEs were not detected in fish, being their levels (rather low) higher in mussels than in oysters: 0.065 and 0.020 mg/kg ww, and 0.037 and 0.012 mg/kg ww, for Ce and La, respectively. A subsequent investigation of the same research group [60] identified patterns and fractionations of REEs, and verified the potential use of REEs as pollution tracers. The concentrations of 16 REEs were measured in various species (brown, green, red) of marine seaweed, which were collected at 3 different sites at the NW Mediterranean Sea. The ΣREEs (in mg/kg dry weight) was 22, 8.5 and 7.9, at sites named S1, S2 and S3, respectively. Seaweeds showed to be a useful tool for biomonitoring REEs, considering that they may be concentrated at higher levels than seawater. Squadrone et al. [61] also measured the concentrations of 10 REEs in 70 samples of different matrices of marine origin collected from the Ligurian Sea (NW Mediterranean Sea). Samples of various species of fish (mullet, redfish, mackerel, and hake) and bivalves (mussels, clams, and oysters) were analyzed. In turn, the levels of REEs were also determined in samples of seaweeds (Chlorophyta, Ochrophyta and Rhodophyta) and zooplankton collected from the Ligurian Sea. The highest ΣREEs was found in seaweed (mean: 12 mg/kg), followed by fish (0.21 mg/kg), bivalves (0.16 mg/kg) and zooplankton (0.12 mg/kg). The ΣREEs concentration in seaweed was significantly higher than the levels detected in fish, bivalves and zooplankton. Taking into account that fish and bivalves are potential sources of human dietary exposure to REEs, the authors suggested to carry out periodical monitoring of their concentrations in order to prevent potential harmful cumulative effects in humans. Regarding the levels of REEs in fish, Squadrone et al. [64] also measured the concentrations of 40 elements (24 trace elements and 16 REEs) in blood, liver, kidney and muscle of spotted dogfish (*Scyliorhinus stellaris*) reared in an Italian aquarium. Although this fish is only of moderate commercial fisheries relevance, human consumption is not negligible in some Mediterranean zones. The mean ΣREEs were 4.1 ± 0.51, 30 ± 1.6, 15 ± 2.0 and 13 ± 1.3 μg/kg in samples of blood, liver, muscle and kidney, respectively, with Sc being the most abundant REE.

On the other hand, Akagi and Edanami [2] measured the levels of 14 REE in five species of bivalves that were collected from three different sites in the Tokyo Bay (Japan). Shells and soft tissues were specifically analyzed. Although there were considerable differences among the levels of the 14 REEs, it was observed that the concentrations of REEs in soft tissues were higher (by an order of magnitude) than those found in shells. In USA, Mayfield and Fairbrother [39] carried out a study focused on characterizing the concentrations of 16 REEs in several freshwater fish species from a large reservoir (6 collection areas) belonging to Washington State. REEs concentrations were measured in tissues and their relationships with tissue type, size, and trophic group were examined. Samples of burbot, kokanee, longnose and largescale sucker, lake and mountain whitefish, rainbow trout, smallmouth bass, sculpin and walleye, were analyzed. The total average level of the measured REEs was 0.243 mg/kg dry weight (dw) (range: 0.014–3.0 mg/kg dw). The benthic feeding species, which are exposed to sediments, showed higher levels of REEs than pelagic omnivorous or piscivorous species. On the other hand, Amyot et al. [3] conducted a study that aimed at determining the background levels and trophic transfer of 15 REEs in natural environments of North America. In that study, samples of abiotic and food web components were collected in 14 Canadian lakes presumably unaffected by REEs mines. Throughout different components of lake food webs, it was found that the individual concentration of the 15 analyzed REEs, as well the sum of REEs were notably related with each other. The sums of REEs were higher in whole body than in muscles for samples of the analyzed species: brown bullhead, creek chub, and white sucker: 32, 40 and 275, respectively. Moreover, the sum of REEs concentrations (median: 20 nmol/g) in benthic invertebrates was approximately 1000 times higher than that found in fish muscle (median: 0.02 nmol/g). The sum of REEs in non-predatory benthic invertebrates (60 ± 69 nmol/g) was significantly higher than those found in predatory benthic invertebrates and in zooplankton: 16 ± 14 and 13 ± 12 nmol/g, respectively. It was concluded that the low concentrations of REEs found in freshwater fish muscle suggest that fish fillet consumption should not mean a significant source of human exposure to REEs in areas that are not affected by mining activities of REEs. In France, Briant et al. [7] measured the levels of 14 REEs in samples of soft tissues of the mussels *Mytilus edulis* and *M. galloprovincialis* and the oysters *Crassostrea gigas*. Samples of these species were collected along the French shoreline, including 31 sampling stations. The sum of REEs concentrations varied among the respective sampling stations, with a range between 0.18 and 10.94 µg/g. The ∑REEs concentrations in mussels and oysters were 0.18–4.05 µg/g and 0.21–10.94 µg/g, respectively. The highest concentrations were found in the Gironde estuary, with the ∑REEs in oysters being up to 10.94 µg/g dw. On the other hand, Reindl and Falkowska [52] determined the concentrations of REEs in muscles of pelagic fish (Baltic herring (*Clupea harengus*) and sardines (*Sardina pilchardus*) from two European regions: the Gdansk Bay (South Baltic Sea) and the Iberian Peninsula (NE Atlantic). These marine species, in various forms (fresh, frozen, processed), are available in the European market. The levels of the examined REEs in the ova and seminal fluid were also analyzed. REEs were detected in muscles of the Baltic herring and sardine, with values of ∑REEs of 0.076 ± 0.047 and 0.191 ± 0.163 mg/kg, for herring and sardine, respectively. The HREEs were the dominant in both analyzed species. For Baltic herring and sardine, the ∑REEs in muscles were lower than those observed in ova and seminal fluid. In Portugal, Figueiredo et al. [22] measured the levels of REEs in mussels (*Mytilus galloprovincialis)* from six locations of the Portuguese coast. The concentrations of ∑LREEs (La, Ce, Pr, Nd, Pm, Sm and Eu) varied between 288 ng/g in Aljezur (the southernmost point on the Portuguese coast) during autumn, and 2160 ng/g in Porto Brandão (south bank of the Tagus estuary) during spring. In turn, the levels of ∑HREEs (Gd, Tb, Dy, Ho, Er, Tm, Yb and Lu) ranged from 54 ng/g in Aljezur during autumn, and up to 230 ng/g in Porto Brandão during spring. In Crimea/Black Sea, Chelyadina et al. [9] measured the concentrations of all the 16 REEs in samples of mussels (*Mytilus galloprovincialis)*. The levels of REEs were analyzed in soft tissues, byssus, and shell liquor of the mussels, which were collected in three sampling locations of Crimea. The average sum of the analyzed REEs in mussels was 0.71 mg/kg dw. In soft tissue and byssus, La and Ce were the dominant elements at the 3 sampling stations, followed by Nd, Sc and Gd. In turn, in shell liquor, the dominant REEs were Tb, Gd and Nd, while La was also among the dominant elements at Stations 2 and 3. The authors concluded that mussel consumption should not possess dietary risk to human health. It was based on considering the values of target hazard quotients according to two estimates of the threshold oral reference dose in terms of REEs intake. In Brazil, recently Santos et al. [55] studied the fate of REEs, their incorporation in biota, and transfer along a food web of the Subaé River, which is one of the main tributaries of the Todos os Santos Bay (northeastern of Brazil), a heavily contaminated estuary. The concentrations of 15 REEs were measured in sediments, suspended particulate matter, as well as in samples of various species of shellfish and fish, which are widely consumed by the local population. The ∑REEs in phytoplankton and zooplankton were 45.7 and 68.5 mg/kg, respectively. These values were considerably lower than those found in bivalves, crustaceans and fish: 6.01, 1.22 and 0.059 mg/kg, respectively. The lowest levels corresponded to fish muscle. It was concluded that although seafood consumption would be unlikely to be an important source of exposure to REEs for humans, taking into account that fish muscle is the main tissue consumed, the levels of REEs in fish and seafood should be periodically monitored. In Iran, and also recently, Bakhshalizadeh et al. [4] determined the levels of 16 REEs in samples of 20 golden grey mullets, which had been caught in Bandar Anzali Coast (south of the Caspian Sea). The levels were compared with the normal values of these elements in the earth’s crust. The ∑REEs was 14,550 pg/g, with a relationship between ∑HREEs and ∑LREEs of 1.53. Although all mullet samples contained much lower levels of REEs than the concentrations of the earth’s surface, it should be highlighted that Tb, one of the scarcest elements in the world, was found to be the third most abundant REEs. It would suggest that the notable Tb presence was the result of rather recent anthropogenic activities.

## Levels of REEs in Vegetables and Fruits

Data on the studies here reviewed which are focused on the determination of REEs in vegetables and fruits are summarized in Table 2. In Hetian Town (Fujian Province, China), Li et al. [37] conducted a study that aimed at measuring the levels of REEs in soil and vegetables collected from farms (6 sampling sites) near mining sites of that place. The health risk of human REEs exposure due to vegetable consumption was evaluated. The levels of the 17 REEs, excluding Sc and Pr, were determined in samples of the following vegetables: Chinese white cabbage, taro, Chinese radish, water spinach, lettuce, long bean, pakchoi, and eggplant. The ∑REEs in vegetables ranged between 0.06 and 64.42 μg/g dw, with a mean of 3.58 μg/g dw. The mean contents of REEs in vegetables collected in the vicinity of the mining sites were significantly higher than those found in samples collected in a control site, which indicated that REEs from rare earth mining was an evident source of REEs pollution. The daily intake of the 8 analyzed vegetables decreased in the following order: taro > water spinach > lettuce > parkchoi > long bean > eggplant > white radish > Chinese cabbage. According to the authors of that study, the consumption of these vegetables would not result in daily intake of REEs that could mean a health risk (safe values: 100–110 μg/kg/day) for adults and children. In a subsequent Chinese study, Zhuang et al. [79] measured the concentrations of REEs in 301 samples of a number of vegetables (edible parts of Chinese cabbage, scallion, white gourd, long bean, eggplant, tomato, potato, towel gourd, pumpkin, red pepper, radish and carrot). Samples were collected from 10 sampling sites of Weishan County: 5 from a mining area in Shandong and 5 control sites. The health risk of human exposure to REEs through vegetable consumption was also assessed. Fourteen REEs (Sc, Pr and Y were excluded) were analyzed. The average concentration of REEs was 66.47 μg/kg, being 94.08 and 38.67 μg/kg the mean levels found for vegetables collected from the mining and control areas, respectively. The difference was statistically significant. Lanthanum, Ce, Pr, and Nd were the most abundant REEs, which accounted for over 91% of total REEs in both mining and control sampling sites. On the other hand, the levels of ∑REEs in the analyzed vegetables decreased in this order: leaf vegetable > taproot vegetable > alliaceous vegetable > bean > solanaceous vegetable > tuberous root vegetable > gourd vegetable. The EDI of the total REEs was 0.69 and 0.28 μg/kg/day, for samples collected in the mining and control areas, respectively. These values were significantly lower than the established EDI (70 μg/kg/day). Although that EDI should not mean health risk in adults, the authors recommended paying attention to the potential risk for children, risk derived of a continuous exposure to low levels of REEs. In the Xinfeng County, Jiangxi province (China), Cheng et al. [10] examined the transfer characteristics from soils to navel oranges of 15 REEs Ce. The effects of these REEs on the internal quality of that fruit were also examined. The concentration of REEs found in the samples of navel orange pulps was rather low (average: 0.341 mg/kg dw; range: 0.106 and 0.829 mg/kg dw). Most contribution corresponded to the LREEs (approximately 84% of the total content of the analyzed REEs), with Ce being the predominant element. The average concentration of REEs found in that study was lower than the food safety limit for REEs set in China two decades ago [42]. It was specifically about 14 times lower. In a more recent study on the same topic, Shi et al. [56] determined the levels of REEs in fruits (288 samples of pome fruits, drupes, citrus, melon fruits, tropical and subtropical fruits, berries, and small fruits) and vegetables (942 samples of solanaceous, brassica, melon vegetables, stem vegetables, root and tuber vegetables, bulb vegetables, fresh legumes and leafy vegetables). Samples were collected near mining areas of China (Bayan Obo in Inner Mongolia, Weishan in Shandong, Maoming in Guangdong and Longnan in Jiangxi), as well as in control sampling points. In fruits, the total concentrations of the sum of Sc, Y, La, Ce, Nd, Pr, Sm, Eu, Gd, Tb, Dy, Ho, Er, Tm, Yb and Lu were 12.90 and 11.89 μg/kg, values found in the mining and control areas, respectively. In turn, the levels of REEs in vegetables were 92.90 and 62.38 μg/kg in mining and control areas, respectively. Regarding the EDI, it was in the range 0.02–0.06 and 0.53–1.22 μg/kg/day for fruit and vegetables. This daily intake was considerably lower than the regular allowable daily intake (60.4 μg/kg body weight) for all gender/age groups of consumers.

**Table 2:** A summary of available information on REE levels in fruits and vegetables

Region/country	Vegetables/fruits	Analyzed REEs	Mean values and/or range	Remarks	Reference
Fujian Province, China	8 species of local vegetables	15	∑REEs, mean: 3.58 µg/g (dw) (0.06–64.42 µg/g)	The daily intake of the analyzed vegetables was safe	Li et al. [37]
Weishan County, China	12 species of vegetables from 10 sampling sites	14	∑REEs, mean: 94.08 and 38.67 µg/kg in vegetables collected in mining and control areas, respectively	La, Ce, Pr and Nd accounted for over 91% of total REEs in mining and control areas	Zhuang et al. [79]
Xinfeng County, Jiangxi Province, China	pulp of navel oranges	15	∑REEs, 0.341 µg/g (dw) (0.106–0.829 µg/g)	LREEs accounted for 84% of the total content of REEs	Cheng et al. [10]
Four mining points (Inner Mongolia, Shandong, Guangdong and Jiangxi), China	228 samples of various fruits and 942 samples of several species of vegetables	16	∑REEs, fruits: 12.90 and 11.89 µg/kg in fruits collected in mining and control areas. In vegetables, 92.90 and 62.38 µg/kg	EDI was in the ranges 0.02–0.06 and 0.53–1.22 µg/kg/day, for fruits and vegetables, respectively	Shi et al. [56]
La Palma, Canary Islands, Spain	bananas collected from an area affected by a volcanic eruption, and control sites	17	∑REEs, 61.4 (peel) and 14.2 (flesh) ng/g. In control sites; 16.2 (peel) and 5.7 (flesh) ng/g	The intake of REEs from consumption of bananas would not exceed 5% of the tolerable daily intake for any of the REEs	Rodriguez-Hernández et al. [53]

In another vein, following a volcanic eruption that occurred in 2012 in the island of La Palma (Canary Islands, Spain), the concentrations of 50 elements were determined in bananas from the affected area [53]. The levels of 17 REEs were determined in that study. The median values of ∑REEs in peel and flesh samples of the fruits collected in the volcano area were 61.4 and 14.2 ng/g, respectively, being 16.2 and 5.7 ng/g the values found to the bananas in the control area. For both peel and for flesh, the differences were significant. Anyhow, these higher levels should not mean a real risk for the consumers. Even the consumption of bananas from the volcano area would be of little relevance in terms of human health. On average, the intake of REEs from consumption of bananas would not exceed 5% of the tolerable daily intake of any of the REEs. On the other hand, in order to gather information about the occurrence of REEs in the daily diet of adults and children, Doulgeridou et al. [15] published a review on potentially toxic REEs, plus thallium and tellurium, in plant-based foods (edible plants). The studies reviewed in that paper have been also included in the current review.

## Mushrooms

It is well-known that mushrooms are effective to bioconcentrate different chemical elements -including also trace elements and REEs- from substrates, in which mycelium lives to develop fruiting bodies. In the scientific literature, we have found several studies that focused on monitoring REEs in edible mushroom species (Table 3). Most of these investigations have been conducted in Poland, Gdańsk. The results of these studies are next summarized. Falandysz et al. [18] determined in composite samples (collected across Poland) of the mushroom *Macrolepiota procera,* (caps and whole fruiting bodies) the levels of 16 REEs. The total concentrations of ΣREEs in caps (13 sites) and whole fruiting bodies (3 sites) were 0.50 and 0.75 mg/kg db (dry biomass), respectively. Cerium was found to be the most abundant REE (mean value in caps: 0.18 ± 0.09 mg/kg db; range: 0.030–0.34 mg/kg db). In contrast, the lowest levels (0.0011 mg/kg db) corresponded to Lu and Tm. According to these authors, the levels of REEs found in *M. Procera* indicated that eating a tasty cap of this mushroom should not mean a health risk for the consumers. In another study conducted by the same research group [19], the multi-elemental composition and associations between 32 trace elements and 16 REEs were assessed. Samples of fruiting bodies (caps and stipes) were collected at 16 sites from northern and central regions of Poland. Among the 48 elements analyzed, the highest levels corresponded to Cd, Hg and Pb found in edible caps of the fruiting bodies. With respect to REEs, as in the survey by Falandysz et al. [18], the concentrations of REEs detected in *M. Procera* should not mean a health risk for the consumers. More recently, Falandysz [17] carried out a survey that focused on extending the data on the levels of 30 metals in samples of the king bolete mushroom, *Boletus edulis* (fruiting bodies). The sum of the concentrations of 14 REEswas calculated. The median value was 0.31 mg/kg dw, with a range between 0.074 and 1.8 mg/kg dw. The concentrations in *B. edulis* of all the elements analyzed in that survey, including the 14 REEs, were considered harmless. In a subsequent study, Falandysz et al. [20] measured the levels of 16 REEs in fourteen composite samples of the fruiting bodies of *B. edulis.* Samples were collected from various locations of Poland. Cerium was the most abundant REEs, followed by La and Nd, with median levels of 95, 51 and 32 μg/kg, respectively. The median REEs concentrations in the caps, stipes, and whole fruiting bodies followed this order: LREEs > MREEs > HREEs. The median concentrations for these three subgroups were the following: 121.4, 12.8 and 5.6 μg/kg dw in caps, 218, 19.1 and 9.2 μg/kg dw in stipes, and 187.7, 17.6 and 8.7 μg/kg dw for the whole fruiting bodies. As for other mushrooms, it was concluded that consumption of *B. edulis*, should not mean any known toxicological risk, even for high-level consumers. Recently, Medyk and Falandysz [40] reported the results of a study that focused on exploring the bioconcentration potential and status of 15 REEs in samples of various species of wild mushrooms and forest topsoil (0–10 cm layer). Eight different species of mushrooms were collected from Poland, Belarus and China. The ƩREEs levels in wild mushrooms were found to be between 15.8 μg/kg dw in caps of *I. badia* and 443 μg/kg dw in *C*. *cibarius*. It was observed that the levels varied between species, with some differences being detected also in REEs concentration from the same species collected at distantly located sampling sites. The same research group [41] carried out a new study whose main purpose was to characterize the presence of REEs in a relatively large sample (22 composites of 2235 specimens) of *Cantharellus cibarius* collected from 22 locations across Poland. In addition, a pooled sample (153 specimens) of *C.minor* collected at Yunnan (China) was also included in that survey. The mean concentration of the sum of 16 REEs in *C. cibarius* was 176 ± 167 µg/kg dw. The pooled sample of *C.minor* from Yunnan contained a higher level of ƩREEs (2072 µg/kg dw) than that of *C.cibarius* collected in Poland. Anyhow, the amounts of the REEs detected in *C.cibarius* mushrooms in that survey would be considered certainly low -and even negligible- from a point of view of food safety.

**Table 3:** A summary of REE levels in mushrooms

Country	Mushroom	Analyzed REEs	Mean values and/or range	Remarks	Reference
Poland	*Macrolepiota procera*	16	ΣREEs in caps: 0.50 mg/kg db ΣREEs in whole fruiting bodies: 0.75 mg/kg db	Cerium was the most abundant REE and lowest levels corresponded to Lu and Tm. Levels of REEs should not mean a health concern	Falandysz et al. [18]
Poland	*Macrolepiota procera*	16	Not specified	Levels of REEs should not mean a health concern for consumers	Falandysz et al. [19]
Poland	*Boletus edulis*	14	ΣREEs: 0.31 mg/kg dw, range: 0.074 – 1.8 mg/kg dw	Concentrations of analyzed elements were considered harmless	Falandysz [17]
Poland	*Boletus edulis*	16	Ce: 95 μg/kg La: 51 μg/kg Nd: 32 μg/kg	Consumption of this mushroom should not mean any toxicological risk, even for high-level consumers	Falandysz et al. [20]
Poland Belarus China	8 species of wild mushroom	15	ƩREEs in *I. badia*: 15.8 μg/kg dw ƩREEs in *C*. *cibarius:* 443 μg/kg dw	Levels varied between species and differences were detected from same species located at distant sampling sites	Medyk and Falandysz [40]
Poland China	*Cantharellus cibarius Cantharellus minor*	16	ƩREEs in *C*. *cibarius:* 176 μg/kg dw ƩREEs in *C*. *minor:* 2072 μg/kg dw	Concentration of REEs in *C*. *cibarius* would be negligible from a point of view of safety	Medyk et al. [41]
Poland	20 mushroom species	13	ƩREEs in above-ground species: 1.39 mg/kg dw ƩREEs in species growing on wood: 1.61 mg/kg dw	No specific remarks	Mleczek et al. [43]
Poland	*Boletus edulis* *Imleria badia* *Leccinum scabrum* *Macrolepiota procera*	15	∑REEs in *B.edulis*: 2.00 mg/kg dw ∑REEs in *I.badia*: 2.43 mg/kg dw ∑REEs in *L.scabrum*: 0.607 mg/kg dw ∑REEs in *M. procera*: 0.593 mg/kg dw	The concentration of REEs in the examined mushrooms increased between 1990 and 2010	Siwulski et al. [58]
Greece	*C. cylindracea* *P.ostreatus*	16	Ce: 31–81 and 33–75 µg/kg for *C.cylindracea* and *P. Ostreatus* respectively La: 13–42 and 15–39 µg/kg for *C.cylindracea* and *P. Ostreatus* respectively Nd: 3.1–53 and 3.4–13 µg/kg for *C.cylindracea* and *P. Ostreatus* respectively Sc: 15–27 and 3.4–13 µg/kg for *C.cylindracea* and *P. Ostreatus* respectively	Consumption of cultivated mushrooms would not be potentially harmful for humans	Koutrotsios et al. [35]
Serbia	*Macrolepiota procera*	16	∑LREEs in S1: 0.47 µg/kg ∑LREEs in S2: 0.93 µg/kg ∑HREEs in S1: 0.032 µg/kg ∑HREEs in S2: 0.041 µg/kg	*M.procera* does not have the ability to accumulate REEs from soild	Vukojevic et al. [70]

Other authors have also determined the levels of REEs in wild (edible) mushrooms species growing in Poland. Mleczek et al. [43] measured the concentrations of platinum group elements (PGEs) and REEs in 20 mushroom species (10 above ground and 10 growing on wood). In *Suillus luteus* and *Tricholoma equestra*, the highest levels of REEs were 5.03 ± 0.50 and 2.18 ± 0.56 mg/kg dw, respectively. In turn, in samples of *Ganoderma applanatum* fruiting (mushrooms growing on wood bodies), the mean REEs concentration was 4.19 ± 0.78 mg/kg dw. In above-ground species and species growing on wood, the mean levels of REEs were 1.39 ± 1.21 and 1.61 ± 0.97 mg/kg dw, respectively. Siwulski et al. [58] determined the contents of 15 REEs in *Boletus edulis*, *Imleria badia, Leccinum scabrum* and *Macrolepiota procera.* The samples of these four edible mushroom species were collected in forests of Poland during the period 1974–2019. The ∑REEs give as mean and range (mg/kg dw) in fruit bodies of these four species were: 2.00 (0.228–4.06), 2.43 (0.879–4.23), 0.607 (0.343–1.71) and 0.593 (0.203–1.46) for *B.edulis*, *I.badia, L.scabrum* and *M.procera*, respectively. It was found that the REEs content in the examined mushrooms increased between 1990 and 2010, decreasing later, mainly in *I.* *badia*.

In Greece, Koutrotsios et al. [35] measured the concentration of 16 REEs in various mushroom production substrates, as well as in the edible fruit-bodies of the species *C. cylindracea* and *P.ostreatus*. As in other studies here reviewed, the REEs detected at higher levels in fruit-bodies was Ce (31–81 and 33–75 µg/kg for *C.cylindracea* and *P. Ostreatus* respectively), followed by La (13–42 and 15–39 µg/kg), Nd (3.1–53 and 3.4–13 µg/kg), and Sc (15–27 and 3.4–13 µg/kg). It was in agreement with the results observed from analysis of substrates. Regarding health risk, the EDI of REEs was estimated to be in the ranges 0.034–0.098 and 046–0.081 µg/kg/day, for *C.cylindracea* and *P.ostreatus,* respectively. The results suggested that consumption of cultivated mushrooms at the REEs concentrations found (even on substrates with a high content of REEs), would not be potentially harmful for humans. In soils from two different geographical sites of Serbia, Vukojevic et al. [70] measured the baseline levels of 16 REEs. Samples were specifically collected in an unpolluted mountain region (S1) and in a forest near the city of Trstenik (S2). The accumulation in the mushroom *Macrolepiota procera* was also estimated. The median values (µg/kg) of the ∑LREEs were 0.21 (S1) and 0.12 (S2), and 0.47 (S1) and 0.93 (S2), in samples of cap and stipe of *M.procera*, respectively. More specifically, the median levels of the ∑HREEs were 0.021 (S1) and 0.012 (S2), and 0.032 (S1) and 0.041 (S2), in cap and stipe samples, respectively. The results of Vukojevic et al. [70] showed that *M.procera* did not have the ability to accumulate REEs from either of the soils, taking into account that BCF values were lower than 1. On the other hand, while preparing the current paper, an interesting critical review of REEs in cultivated macrofungi has been published by Falandysz et al. [21]. These authors have concluded that a significant proportion of the previously reported data show unexplained anomalies in occurrence patterns. This might be unsuitable for assessing human health risk derived from exposure to REEs, as well as to evaluate potential increasing trends of REEs contamination in foodstuffs (Table 4).

**Table 4:** A summary of REE levels in other foodstuff

Country	Foodstuff	Analyzed REEs	Mean values and/or range	Remarks	Reference
Jamaica	Fruits, legumes, leafy and root vegetables and other root crops	5	Ce in “callaloo”: 0.24 mg/kg La: 0.004 – 0.061 mg/kg Eu: 0.3 – 1.09 mg/kg Sc: 0.4 – 3.4 mg/kg Sm: 0.6 – 4.6 mg/kg	It would be unlikely that dietary exposure to these REEs lead to a public health risk	Howe et al. [29]
China	Fresh vegetables, cereals, fresh meat, eggs, and aquatic products	16	∑REEs: 0.2060 mg/kg	No specific remarks	Jiang et al. [31]
China	Cereals	14	∑REEs: 55.79 μg/kg	Results showed that La, Ce, Pr and Nd were the most abundant REEs. The EDI of the total REEs was lower than the allowable daily intake	Zhuang et al. [80]
Italy	Honey	16	∑REEs: 9.1 – 65 μg/kg	No specific remarks	Squadrone et al. [62]
Italy	Honey	16	∑REEs: 6.0 – 14 μg/kg	Consumption of honey should not mean a health risk	Squadrone et al. [63]
Jordan	Honey	15	∑REEs: 145 ± 157 μg/kg	Concentrations in honey were found to be much lower than their guideline limits, indicating their safety for human consumption	Tahboub et al. [65]
China	Tea	16	∑REEs in black tea: 2.960 mg/kg ∑REEs in green tea: 2.500 mg/kg ∑REEs in oolong tea: 3.870 mg/kg ∑REEs in dark tea: 2.955 mg/kg	Highest content of REEs in tea corresponded to Ce	Wang et al. [71]
-	Tea	16	Not provided	Exposure to REEs through tea consumption should pose a negligible risk to the consumer	Kowalczyk et al. [36]
Australia	Rice	16	∑REEs: 0.009 – 128.2 μg/kg	Differences detected among samples of the selected countries could be due to different agricultural practices, geological and topographical variation	Imran et al. [30]
Italy	Plant feed, fruits, honey and wildlife livers	10	Highest ∑REEs found in plant feed (1.8 mg/kg), wildlife livers (0.043 mg/kg), fruits (0.0088 mg/kg) and honey (0.0078 mg/kg)	No specific remarks	Squadrone et al. [61]
Sweden	Cereal products, seeds, potato products, vegetables, algae and algae products and new food items	14	Refer to the original article	No specific remarks	Kollander et al. [34]
Spain	Ready-to-eat baby food	17	∑REEs: 5.9 – 16.1 ng/g fresh product	Children at the 97.5th percentile of consumption reached the maximum tolerable daily intake	Henriquez-Hernández et al. [27]
Greece	Fava Santorinis and non-Fava Santorinis	12	REEs Fava Santorinis: < LOD – 6464 ng/kg REEs non-Fava Santorinis: 284 – 32,031 ng/kg	No specific remarks	Drivelos et al. [16]
Italy	Flour	15	∑REEs: 0.0226—3.0573 mg/kg	Cricket flour contains significantly higher amounts of REEs compared to other flours. Therefore, exposure to REEs is more plausible when cricket flour is consumed	Ruffolo et al. [54]

## Other Foodstuffs

Howe et al. [29] analyzed the concentrations of 27 elements in samples of various food that were collected from farms and markets in central Jamaica. The following food groups were included: fruits, legumes, leafy and root vegetables, as well as other root crops (being potatoes the item most consumed of this group). Among the 27 analyzed elements, Ce, Eu, La, Sc and Sm, were included. It was noted that for Ce, most samples were below its limit of detection. The highest Ce concentrations were found in “callaloo” and red kidney beans being 0.24 and 0.006 mg/kg, respectively. For the rest of analyzed REEs, the concentrations showed these ranges: 0.004 (fruits)-0.061 mg/kg (vegetables leafy); 0.3 (vegetables leafy)-1.09 µg/kg (fruits); 0.4 (fruits)-3.4 µg/kg (vegetable roots) and 0.6 (fruits)-4.6 µg/kg (vegetables leafy), for La, Eu, Sc and Sm, respectively. The authors stated that it would be unlikely that dietary exposure to these 5 REEs might lead to a public health risk. In China, Jiang et al. [31] determined the levels of 16 REEs in samples of fresh vegetables, cereals, fresh meat, eggs, and aquatic products (mollusks, crustaceans, and marine and freshwater fish). The average contents of Dy, Ce and La were between 0.0524 and 0.0289 mg/kg. The mean levels of Sc, Y, Er, Nd, Gd and Pr were between 0.0184 and 0.0082 mg/kg, while those of Sm, Yb, Ho, Eu, Tb, Tm and Lu were in the range 0.0038–0.0012 mg/kg. The mean concentration of the total analyzed REEs was found to be 0.2060 mg/kg. In general, the 16 REEs were detected at low levels in all the examined foodstuffs. Also in China, Zhuang et al. [80] measured in cereals the levels of 14 REEs. Samples were collected in mining and control areas in Weishan County. More specifically, near to a large-scale REEs mining site in Shandong. The potential health risk derived of the REEs exposure through cereal consumption was also assessed. The average concentration of ΣREEs for the 327 analyzed samples was 55.79 μg/kg, while for the samples of cereals collected from the mining and control areas, the mean concentrations were 74.22 and 47.83 μg/kg, respectively. It was found that La, Ce, Pr and Nd were the most abundant REEs, accounting for over 90% of the total REEs for the mining area. The EDI of the total REEs was considerably lower than the allowable daily intake (70 μg/kg bw).

Some data on the concentrations of REEs in honey are also available in the scientific literature. Squadrone et al. [62] conducted a survey that focused on assessing the occurrence of a number of trace elements and 16 REEs in honey from various countries. Another objective of that study was to evaluate if the concentrations of these elements in honey might be permissible for human consumption. All the elements were determined in samples of multi-floral honey from the following countries/regions: Italy (n = 40), Balkans (n = 10), Kazakhstan (n = 10), Tanzania (n = 5) and South America (n = 10). The ΣREEs in multi-floral honey followed this decreasing order: Tanzania (65 μg/kg) > Italy (11 μg/kg) > South America (9.8 μg/kg) > the Balkans (9.3 μg/kg) > and Kazakhstan (9.1 μg/kg). The LREEs/HREEs ratio ranged between 0.28 (Italy) and 2.5 (Tanzania). The same research group [63], also determined in monofloral and multifloral honey from Piedmont (NW Italy) the levels of 24 trace elements and the same 16 REEs also measured by Squadrone et al. [62]. The safety of the consumption of the analyzed honey was also assessed. Ninety-one samples of acacia (n = 26), chestnut honey (n = 18), linden honey (n = 15), rhododendron honey (n = 14) and multifloral honey (n = 18) were collected and analyzed for the 16 REEs. The sum of REEs were 6.6, 12, 11, 14 and 6.0 μg/kg, for acacia, chestnut honey, linden honey, rhododendron honey and multifloral honey, respectively. The highest concentrations of REEs were found in chestnut honey, while the lowest levels corresponded to acacia and rhododendron honeys. Based on the results (not only for REEs, but also for the analyzed trace elements) of that survey, and considering the nutritional value of the analyzed honey, their consumptions are recommended and they should not mean any potential health risk. In Jordan, Tahboub et al. [65] conducted a survey that aimed at determining the concentrations of 46 elements in 18 imported samples and 12 local samples of honey (monofloral and multifloral). Among the 45 analyzed elements, 15 REEs were included. The highest levels (in decreasing order) corresponded to Ce > Nd > La > Pr > Sm. The total mean of ∑REEs was 145 ± 157 μg/kg (range: 41–489 μg/kg), with the mean levels being 203 ± 165 and 98 ± 113 μg/kg, for local and imported honey samples, respectively. For all analyzed elements, including those potentially toxic and the 15 REEs, the concentrations in honey were found to be much lower than their guideline limits, indicating their safety for human consumption. In China, Wang et al. [71] measured the levels of 16 REEs in 3011 tea samples of four types (black tea, green tea, oolong tea and dark tea). Samples were obtained in the main tea producing areas in different provinces of China. The highest mean levels of ∑REEs (mg/kg) were 2.960, 2.500, 3.870 and 2.955 in black tea (Henan province), green tea (Shanxi province), oolong tea (Guizhou province) and dark tea (Hunan province). The tea leaves accumulated mainly LREEs rather than HREEs. In general, the highest content of REEs in teas corresponded to Ce, regardless of the province of collection. The most abundant REEs followed the order Ce > La > Sc > Nd > Y in black and green teas, the trend Ce > Y > Sc > Nd > La in oolong tea, and Ce > Y > La > Nd > Sc in dark tea. Recently, Kowalczyk et al. [36] measured in samples of teas the concentrations of 16 REEs. The levels of Sb, Ba, B, Li, Te, Tl and V were also determined in the same samples. The human health risk derived of the tea consumption was also assessed. Due to the lack of substantial information on the toxicity of most REEs, the related uncertainty was considered as high. Lanthanum, Ce and Y were found to constitute 60% of total REEs contamination in teas, being the contribution from the rest of REEs considerably lower. Based on these findings, Kowalczyk et al. [36] concluded that exposure to REEs through tea consumption should pose a negligible risk to the consumers. No adverse effects should be expected even for high tea consumers. Recently, Imran et al. [30] determined the levels of 16 REEs in 64 samples of rice grown in Australia and imported rice from various countries (India, Italy, Pakistan, Sri Lanka, Thailand, Vietnam and USA). The average levels of REEs were 0.013–2.974, 0.012–3.113 and 0.009–0.919 μg/kg in Australian, Thailand and Vietnamese rice samples, respectively. The highest average levels of REEs corresponded to samples from Pakistan (0.299–128.2 μg/kg), India (0.063–20.574 μg/kg) and Sri Lanka (0.022–11.522 μg/kg). Individually, the concentrations of LREEs (Sc, La, Ce, Nd) in rice were higher than those of HREEs. According to the authors, the differences detected among samples of the selected countries could be due to different agricultural practices, geological and topographical variation, as well as the use of fertilizers with different REEs composition. The health risk for the Australian population due to rice consumption was not assessed in that study. On the other hand, the bioaccumulation of the REEs Sc, Ce and Eu (plus Hf and Ta, as well as other trace elements) was evaluated in oats in barley grown in soils of Russia, soils with different characteristics and level of contamination [57]. Soil samples for that survey were collected at 3 different sites (named S1, S2 and S3) in St. Petersburg. The plants were grown in these soils, which differed in texture, pH, and concentrations of exchangeable cations, and also important, in the levels of contamination. The concentrations of Sc in roots ranged between 0.11 and 0.37 mg/kg in oats, and between 0.15–0.23 mg/kg in barley, respectively, being the levels of Ce 1.9–3.8 mg/kg and 1.7–1.8 mg/kg in samples of oats and barley. The concentrations of Eu ranged between 0.04 and 0.08 mg/kg in oats, and between 0.009 and 0.017 mg/kg in barley. The values found in roots were significantly higher than those detected in the leaves of sample of oats and barley. In a previous study conducted in Italy by Squadrone et al. [61], the levels of 10 REEs were measured in samples of plant feed, fruits, honey and wildlife livers. Specifically, 30 samples of plant feed (bran, oat, forage, wheat, and barley), 30 samples of fruits (apples, strawberries, peaches and apricots) and 30 samples of honey were collected in the Piedmont Region (NW Italy). Fifteen liver samples of wild species (boar, roe deer, deer, been sparrow hawk, tawny owl and crow) were also collected in that region. These samples were from animals found dead by local veterinarian services. The highest levels (means in mg/kg) of the sum of REEs were found in plant feed (1.8), followed by wildlife livers (0.043), fruits (0.0088) and honey (0.0078). Among all the analyzed samples, fruits and honey contained very low REEs levels, while plant feed, -mainly represented by raw cereals- included the highest REEs levels, which might mean a major source of exposure. Recently, Kollander et al. [34] reported the concentrations of 74 elements in traditional and new food varieties of the Swedish market. Fourteen REEs were analyzed in that survey. The food groups included cereal products, seeds, potato products, vegetables, algae and algae products, as well as “new” food items (vegetarian protein products, quinoa, teff flour, chia and psyllium seeds). This study measured levels of elements that are not routinely analyzed in foods, and which are not requested within the EFSA call for data. Due to the enormous quantity of data contained in that survey, we have not here reported the specific concentrations for all the analyzed elements including the 14 REEs. For detailed and extensive information, we refer to Kollander et al. [34]. Recently, Henriquez-Hernández et al. [27] determined the content of 38 elements in 159 samples of ready-to-eat baby food sold in Spain. Among the analyzed elements, 17 REEs were included. The median concentrations (ng/g fresh product) of ΣREEs in ready-to-eat puree for babies were 7.3 (fruit purees), 6.2 (chicken purees), 9.6 (fish purees) and 10.1 (beef purees) in major name brands. In store brands, the median levels were 5.9 (fruit purees), 12.1 (chicken purees), 8.0 (fish purees) and 16.1 (beef purees). For risk assessment, the analysis was carried out for two different exposure scenarios: a) children, who were at the average consumption of the foods considered, and b) children, who were at the 97.5th percentile of consumption. In the first scenario, no risk associated with the intake of REEs was found, while in the second scenario, the maximum tolerable intake (according to data reported by other authors) was reached.

In turn, Ruffolo et al. [54] determined the concentrations of 15 REEs in different types of flour (wheat, whole wheat, buckwheat, rice, coconut, kamut, semolina, turmeric, oat and cricket) marketed in Italy. Mean ΣREEs were ranged between 0.0226 to 3.0573 mg/kg. Cricket flour contains significantly higher amounts of REEs compared to other flours. Therefore, exposure to REEs is more plausible when cricket flour is consumed. Finally, Drivelos et al. [16] measured the concentrations of 12 REEs in non-fava Santorinis and in Fava Santorinis, a protected designation of origin of yellow split pea species that are only grown in the island of Santorini (Greece). Results showed that mean levels of REEs in fava Santorinis were ranged between < LOD for Ho and 6464 ng/kg for Y, while for non-fava Santorinis the levels were higher (range between 284 ng/kg for Ho and 32031 ng/kg for Ce).

## Human Dietary Exposure to REEs and Risk Assessment

In recent decades, due to the widespread use of REEs in electronics industry, lasers, advanced materials, superconductors and medicine, among many others, the demand for REEs has dramatically increased. Consequently, this has led to an environmental release of these elements, which has been/is being clearly detected in recent years [26, 47, 49, 76]. Therefore, human exposure to REEs, which could have been more or less irrelevant until recent decades, in the next few years could be of concern [45, 75]. It is well established that for non-occupationally exposed individuals, the diet is the most important route of exposure to metals [5, 23]. In relation specifically to REEs, in recent years some human dietary exposure to these elements have been conducted in China. The results are now available in the scientific literature. Dai et al. [11] determined the exposure to REEs in the Chinese resident diet through a market-based study. Samples of cereals, beans, potatoes, leafy vegetables, root vegetables, melon vegetables, legumes, edible fungi, pork, beef, mutton, poultry, eggs, pure milk, mixed animal fats, fish, shrimp, shellfish and cephalopods, were collected from 33 cities of China, and the concentrations of 16 REEs were measured. The average concentration of the sum of REEs was 64.95 μg/kg ww (range: 2.12–809.68 μg/kg ww). The highest ΣREEs (605.46 μg/kg) corresponded to shellfish samples, while the lowest ΣREEs level was detected in milk samples (18.43 μg/kg). The EDI of REEs was estimated for 5 Chinese regions of China, being 0.54 μg/kg/day the average intake of REEs via food consumption. The maximum EDI was found in Midland (0.90 μg/kg/day). The highest health risk of dietary exposure to REEs corresponded to cereals intake, with a contribution to the total EDI -in the 5 regions- between 45.44% and 69.78%. It was followed by vegetable and aquatic animals’ intake. In contrast, the risk due to the consumption of milk, eggs, and beans would be relatively lower, contributing approximately with a 10% of the total EDI. In general, the health risk of REEs detected in that study was considered as acceptable. A similar survey was also conducted by Yang et al. [74]. This study aimed at investigating the levels and patterns of 16 REEs (Pm was not included) in a number of food samples of 11 main categories, obtained in 31 Provinces of China, as well as to assess the dietary intake of REEs by the Chinese population. The mean concentrations of Ce, La, Y and Nd (0.11, 0.06, 0.05 and 0.04 mg/kg, respectively) in various food categories were higher than those of the rest of REEs. In all food samples of the 11 categories, it was found that the mean ΣREEs was in the range 0.04–1.41 mg/kg. In turn, the daily mean dietary exposure of the ΣREEs was 1.62 μg/kg body weight (bw) in the general Chinese population, with a range of 1.61–2.80 μg/kg bw for the different sex/age groups. Among the 16 analyzed REEs, the highest exposure levels corresponded to Ce, La and Y. The highest mean dietary exposure to these 3 REEs was 0.54, 0.31 and 0.17 μg/kg bw, respectively. The sum of the exposures of these 3 REEs accounted about 63% of the total exposure to the 16 analyzed REEs. On the other hand, the most important contributors to the dietary intake of REEs were vegetables and grains, with percentages of 45.3% and 28.6%, respectively. Although the levels of REEs in tea were the highest among all of the food categories, taking into account that the consumption of this product was comparatively very low, tea resulted in a low contribution (3.6%). Recently, Zhao et al. [78] determined the concentrations of 16 REEs in samples of the most representing foods from the Bayan Obo mining area (Inner Mongolia, China). These samples were randomly selected from local wholesale agricultural markets. The potential health risk of dietary exposure to these 16 REEs was also assessed. The following samples were analyzed for the content of REEs: 140 grain samples, 309 meat and aquatic samples, 153 vegetable samples, 15 fruit samples, 39 dairy samples, and 21 egg samples. The order of REEs concentrations in all food samples was La > Ce > Nd > Y > Pr > Sc > Gd > Eu > Tm > Tb > Er > Sm > Dy > Yb > Ho > Lu. Among all the food analyzed, meat and aquatic samples showed the highest REEs levels, followed by grains, fruits, eggs, vegetables and dairy products. La and Ce were the most concentrated REEs in the analyzed samples. Regarding human dietary exposure, cereals showed the highest contribution of REEs, meaning a major source of exposure. The mean EDI of the total REEs was 0.275 µg/kg/day (range: 0.116–0.512 µg/kg/day). This mean value is below the recommended safe daily average intake of REEs (70 μg/kg/day) [11]. Also in China, Zhuang et al. [81] analyzed the concentrations of 16 REEs in several agricultural products, which were collected in the vicinity of a large REEs mining area in Shandong Province. Their dietary intake by residents was subsequently estimated. Samples of wheat, maize, dry beans, vegetables, fruits and eggs were analyzed in that survey. Considering all samples, the mean value of total REEs was 286.96 μg/kg, with the content of LREEs being 270.18 μg/kg, a value that corresponded to more than 94% of the total REEs. Among the LREEs, Ce, La, Nd and Pr were the dominant elements. The content of HREEs was 16.77 μg/kg. Wheat, leafy vegetables, and allium vegetables had the highest content of REEs, being the main sources of dietary exposure to REEs. In contrast, the lowest contents of REEs were found in melons vegetables, root vegetables, fruits, and eggs. The EDI of REEs was 4.20 μg/kg bw/day. This was a low level that did not exceed the acceptable daily intake. There were scarce differences in the intake of REEs for the different age/gender groups of population.

## Conclusions

Due to the expanding use of REEs in a notable number of high-tech applications, these elements are currently being of considerable importance for the modern world economy. A direct consequence of the increased use of REEs also means that they are being now an emerging pollutants. Considering its current extensive use, it may cause an increasing risk of environmental contamination [6, 26]. Consequently, there is also a potential increased human exposure to REEs, elements on whose toxicity the available information is rather scarce [8, 28, 46]. It is more than probable that the use of REEs will continue increasing in the next years. Therefore, exhaustive investigations aimed at establishing the human exposure and potential adverse effects of these elements are clearly required. In this paper, the results of the studies that focused mainly on determining the levels of REEs in foodstuffs have been reviewed. As indicated above, the diet is the main route of exposure to metals for non-occupationally exposed subjects. Thus, to know the current dietary intake of REEs is an important first step. The results of most studies here reviewed do not suggest any relevant health risk for the consumers of the analyzed freshwater and marine species of higher consumption or derived from the intake of a number of vegetables, fruits, mushrooms and other various foodstuffs (honey, tea, rice, etc.). However, it should be expected that the environmental concentrations of REEs will be increased with the increased use of these elements, which might lead to increased levels of contamination in fish, vegetables, fruits, etc. Consequently, a continued monitoring of the levels of REEs in food is strongly suggested. This monitoring is being already conducted for well-known toxic elements such as arsenic, lead, cadmium and mercury. To assess periodically the potential health risk of the dietary exposure to REEs is, logically, another important issue. For this, it is essential to know/update the toxic effects of the REEs, for which information is not currently particularly abundant.

## Declarations

### Credit Author Statement

Neus González: Writing – original draft.

José Luis Domingo: Conceptualization; Writing – review & editing.
